# Different electrophysiological characteristics of cavo‐tricuspid isthmus dependent atrial flutter guided by robotic magnetic navigation in patients with and without prior cardiac surgery

**DOI:** 10.1002/clc.24098

**Published:** 2023-07-25

**Authors:** Qingzhi Luo, Yun Xie, Yangyang Bao, Yue Wei, Changjian Lin, Ning Zhang, Tianyou Ling, Kang Chen, Wenqi Pan, Liqun Wu, Qi Jin

**Affiliations:** ^1^ Department of Cardiovascular Medicine Ruijin Hospital, Shanghai Jiao Tong University School of Medicine Shanghai China

**Keywords:** atrial flutter, cardiac surgery, catheter ablation, cavotricuspid isthmus, robotic magnetic navigation

## Abstract

**Backgroud:**

Cavo‐ tricuspid isthmus dependent atrial flutter (CTI‐ AFL) is a common atrial arrhythmia in patients with prior cardiac surgery (postsurgical AFL) and without prior cardiac surgery (nonsurgical AFL). However, there is only limited data regarding the eletrophysiological differences between the CTI‐ AFL in the postsurgical patients and the nonsurgical patients.

**Hypothesis:**

We aimed to investigate the differences in clinical and electrophysiological characteristics between the postsurgical group and nonsurgical group and to evaluate the acute and long‐term outcomes after ablation guided by robotic magnetic navigation (RMN) in both the groups. Methods Fourty‐two consecutive patients with nonsurgical AFL and 21 with postsurgical AFL were retrospectively analyzed in our center. Electrocardiographic (ECG) analysis and three‐dimensional electrophysiological study were performed in all the patients.

**Results:**

The results revealed that only 55.6% of postsurgical patients with proven counterclockwise (CCW) AFL presented with a typical ECG suggesting this mechanism. In contrast, 86.1% of nonsurgical patients demonstrated a typical ECG pattern for CCW AFL. In addition, we employed a reverse “U‐curve” to facilitate radiofrequency delivery when ablating near the inferior vena cava ostium in the present study. Compared with the nonsurgical group, electroanatomical mapping showed the mean AFL cycle length was significantly longer (253.3 ± 40.4 vs. 234.1 ± 24.2 ms, *p* = 0.03) and the right atrium volume was larger (114.8 ± 26.0 vs. 97.5 ± 19.1 mL, *p* = 0.004) in the postsurgical group. Additionally, the procedural time (75.9 ± 21.3 vs. 61.6 ± 26.6 minutes, *p* = 0.03) and ablation time (53.0 ± 21.4 vs. 36.7 ± 25.6 minutes, *p* = 0.02) are much longer in the postsurgical group. However, the navigation index in the postsurgical group was significantly smaller (0.35 ± 0.08 vs. 0.43 ± 0.13, *p* = 0.01). Moreover, the acute and long‐term success rates were comparable between the two groups.

**Conclusions:**

Catheter ablation of CTI‐AFL with and without prior cardiac surgery guided by RMN are associated with high acute and long‐term success rates, despite the procedural and ablation times are much longer in the postsurgical patients. However, ECG characteristics of the tachycardia may be misleading as they are more often atypical in patients after cardiac surgery.

## Graphical abstract

Catheter ablation of cavo‐tricuspid isthmus dependent atrial flutter with and without prior cardiac surgery guided by robotic magnetic navigation are associated with high acute and long‐term success rates, despite the procedural and ablation times are much longer in the postsurgical patients. However, electrocardiographic characteristics of the tachycardia may be misleading as they are more often atypical in patients after cardiac surgery.

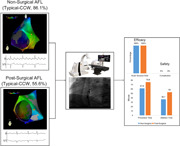

## 1 INTRODUCTION

Cavo‐tricuspid isthmus dependent atrial flutter (CTI‐AFL) is a common atrial arrhythmia in clinical practice and has been well studied. The electrophysiological mechanism of CTI‐AFL in patients without previous cardiac surgery is macroreentrant tachycardia around the tricuspid annulus (TVA) in the right atrium (RA) in a clockwise (CW) or counterclockwise (CCW) direction.^1^ Meta‐analysis shows that catheter ablation of CTI‐AFL is regarded as a procedure with high long‐term success and low complication rates.^2^ In patients subjected to previous cardiac surgery and RA atriotomy, the incidence of complex atrial tachycardias (ATs) increases at subsequent follow‐up, in which mechanisms related to surgical incisions, atrial dilatation, and structural remodeling with conduction slowing create the substrate for macroreentry.^3^, ^4^, ^5^ The most common postsurgical ATs in these patients are CTI‐AFL, scar‐related ATs, atrial fibrillation (AF) and less commonly focal ATs.^6^, ^7^ However, there is only limited data regarding the electrophysiological differences between the CTI‐AFL in the nonsurgical patients and the postsurgical patients. Thus, the aims of the present study were (i) to investigate differences in clinical and electrophysiological characteristics of CTI‐AFL between the postsurgical and nonsurgical groups and (ii) to evaluate the acute and long‐term outcomes after ablation guided by robotic magnetic navigation (RMN) in both the groups.

## 2 METHODS

### 2.1 Patient selection and data collection

In this retrospective single center study, a total of 63 patients, including postsurgical CTI‐AFL (*n* = 21) and nonsurgical CTI‐AFL (*n* = 42), underwent catheter ablation of CTI guided by the RMN system from 2018 to 2021. All the patients underwent initial evaluation including a medical history, baseline demographics, electrocardiographic (ECG) findings and echocardiography data. Details of the surgical repair were obtained from hospital records for the postsurgical group. Ethical approval for this study was granted by the local institutional committee and informed written consent were obtained from all the patients before the study.

### 2.2 ECG analysis

**Figure 1:**
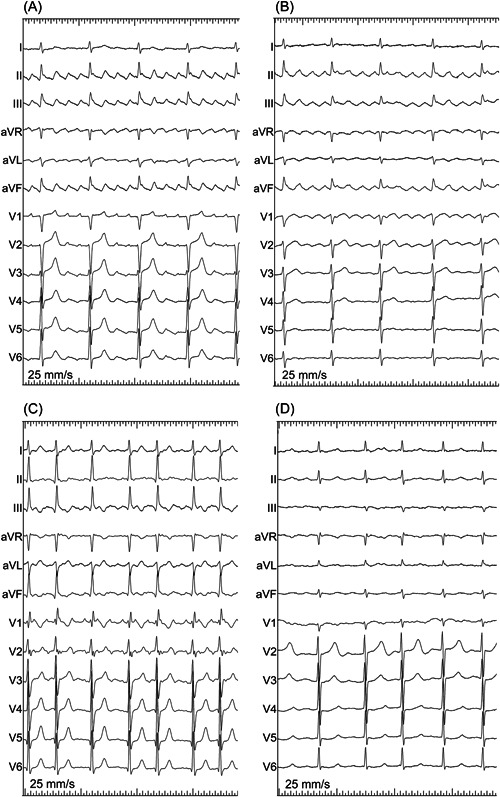
(A) Presents a typical ECG example of CCW‐AFL: the flutter waves are characteristic sawtooth pattern, negative in the inferior leads, positive in lead V1; (B) presents a typical ECG example of CW‐AFL: the flutter waves are positive in the inferior leads without isoelectric line, negative in lead V1; (C) presents an atypical ECG example of CCW‐AFL: the flutter waves are not sawtooth pattern in the inferior leads, differ considerably, positive in lead V1; (D) presents an atypical ECG example of CW‐AFL: the flutter waves are low and flat in the inferior leads with significant isoelectric line, negative deflections in lead V1. AFL, atrial flutter; CCW, counterclockwise; CW, clockwise; ECG, electrocardiographic.

Twelve‐lead ECG of all the patients were analyzed by two independent and experienced investigators. Flutter wave morphologies were assessed in the inferior leads and in lead V_1_. In this study, typical flutter waves of CCW‐AFL were defined as having a sawtooth pattern, negative flutter waves without isoelectric lines in the inferior leads, and positive in lead V_1_; and in CW‐AFL, typical flutter was defined as being positive in the inferior leads and negative in lead V_1_ (Figure 1). Isoelectric lines in atypical flutter were defined as continuous lines between distinct flutter waves.

### 2.3 Electrophysiological mapping and ablation

All antiarrhythmic drugs, with the exception of beta‐blocking agents, were discontinued at least five half‐lives before the electrophysiological study. All the patients were uninterruptedly anticoagulated at the time of the procedure and transesophageal echocardiograms were performed to exclude atrial thrombosis. Standard multielectrode catheters were positioned in the coronary sinus and right ventricular apex (RVA) through the left femoral vein for recording and pacing. A 3.5 mm tip irrigated magnetic catheter (NaviStar™ RMT ThermoCool™, Biosense Webster Inc.) was advanced into the RA using an 8.5‐French long sheath (SR0, St. Jude Medical Inc., St. Paul, MN) via the right femoral vein for three‐dimensional (3D) electroanatomical mapping and ablation guided by the CARTO (Biosense Webster Inc.) and the RMN Niobe ES system (Stereotaxis Inc.). In case of sinus rhythm or cessation of the AFL during mapping, induction of the tachycardia was attempted with programmed atrial stimulation with or without isoproterenol infusion. Both activation and voltage mapping were constructed during AFL to identify the underlying mechanism and select target sites for ablation. Entrainment pacing were performed in the isthmus, roof, and lateral wall of the RA respectively to confirm the involvement of the entire TVA. Good entrainment pacing was defined as concealed entrainment and a short postpacing interval (PPI < 30 ms). Radiofrequency (RF) was delivered at the CTI in a linear fashion with a power control mode (30−35 W, 43°C maximum temperature, and irrigation rate of 17−30 mL/min, 30 s applied at each site). Procedural success was defined as follows: the interruption of the tachycardia during RF delivery and bidirectional conduction block along the CTI validated with pacing and mapping maneuvers as previously described.^8^

In routine practice, the ablation catheter is often unstable when ablating in proximity to the inferior vena cava (IVC) and additional RF lesions may be needed to achieve bidirectional CTI block. Hence, in our center we usually advance the sheath more distally along with the magnetic catheter, and then employ a reverse “U‐curve” to facilitate RF delivery when ablating near the IVC ostium (Figure 3).

### 2.4 Definition of procedural parameters of RMN guided ablation

Procedure time was defined as the total time from Navigant™ “Open Procedure” to Navigant “Close Procedure” (in minutes). Clinical start time was annotated as the earliest of either the time at which the catheter was registered in the CARTO™ 3D mapping system or the time of first applied magnetic field. Clinical time was calculated as the time elapsed between clinical start time and the latter time of either last RMN applied field or the end of the last RF ablation. Mapping time was the time interval from clinical start time to first burn. Total X‐ray time was defined as the number of minutes the fluoroscopy beam was activated. RF applications and RF time reflected the total sum of the number and minutes of ablation burns during the procedure, respectively. Ablation time was calculated as the time difference between the first and last RF application times. Navigation index, defined as the ratio of total RF delivered (in minutes) to the time elapsed from first burn to last burn, was utilized to indicate the efficiency of RMN‐guided ablation in this study. The higher the navigation index, the greater percentage of procedure time was spent delivering RF treatment versus locating or navigating to desired RF treatment locations.

### 2.5 Follow‐up

Following AFL ablation, all the patients were generally discharged home on postprocedure day one unless peri or postprocedure complications dictated further in‐patient monitoring and evaluation. Patients were placed on rivaroxaban or edoxaban for a period of at least 2 months following AFL ablation and all antiarrhythmics were discontinued. Patients were scheduled for routine follow‐up at 1, 3, 6, and 12 months after ablation and then yearly, at which time a determination of recurrence of arrhythmia was made based on symptoms and 12‐lead ECG or Holter monitoring. Only CTI‐AFL episodes were considered as true recurrences.

### 2.6 Statistical analysis

Quantitative variables with normal distribution were given as mean ± SD and were compared using *t*‐tests. Variables that did not follow normal distributions were compared using the Mann–Whitney test. Categorical variables were given as counts or percentage and were compared using the *χ* ^2^ test or Fisher's exact test when approximate. For all tests, two‐tailed *p* < 0.05 were considered statistically significant. Analyses were performed with SPSS 20.0 (IBM SPSS Statistics).

## 3 RESULTS

### 3.1 Patients clinical characteristics

From 2018 through 2021, a total of 63 patients who underwent electrophysiology study and CTI ablation guided by RMN at our institution were included. The postsurgical AFL group comprised 21 patients (55 ± 13 years, 48% male) who had undergone cardiac surgery. The mean time interval between the first symptomatic episode of AFL and cardiac surgery was 65 ± 92 months (range from 1 months to 18 years). A similar RA free‐wall approach was used in all the postsurgical patients, including four patients with atrial septal defect (ASD) repair (one with ASD repair + TV annuloplasty), three with mitral valve replacement (MVR) + TV annuloplasty, five with MVR, two with aortic valve replacement, two with coronary artery bypass grafting (CABG), three with CABG + MVR, and two with left myxoma resection. The Nonsurgical AFL group comprised 42 patients (62 ± 12 years, 83% male). The baseline characteristics of our study cohort are summarized in Table 1. The postsurgical AFL group were significantly older (*p* = 0.03), while the nonsurgical AFL group were more often male (*p*＜0.001). There were no significant differences between the two groups in terms of BMI, comorbidities, and echocardiography parameters including left atrium diameter, left ventricle end‐diastolic diameter, left ventricle ejection fraction, and CHA_2_DS_2_‐VASc score.

**Table 1:** Patients baseline characteristics.

Parameters	Postsurgical group (*n* = 21)	Nonsurgical group (*n* = 42)	*p* Value
Gender (male)	47.6% (10/21)	83.3% (35/42)	0.003
Age (years)	55.7 ± 12.9	62.9 ± 12.3	0.03
BMI	24.5 ± 2.8	24.5 ± 3.2	0.91
Comorbidities			
Heart failure	14.3% (3/21)	21.4% (9/42)	0.49
Hypertension	28.6% (6/21)	26.2% (11/42)	0.84
Coronary artery disease	23.8% (5/21)	9.5% (4/42)	0.11
Diabetes mellitus	23.8% (5/21)	19% (8/42)	0.65
Echocardiographic data			
LAD (mm)	43.8 ± 5.7	41.5 ± 4.8	0.10
LVEDD (mm)	48.9 ± 5.0	48.9 ± 5.7	0.98
LVEF (%)	58.5 ± 9.2	58.9 ± 11.1	0.86
CHA_2_DS_2_‐VASc sore	2.0 ± 1.7	1.8 ± 1.4	0.46

Abbreviations: BMI, body mass index; LAD, left atrium diameter; LVEDD, left ventricle end‐diastolic diameter; LVEF, left ventricle ejection fraction.

### 3.2 Electrophysiological mapping and ECG characteristics

Data on electrophysiological mapping and ECG characteristics are shown in Table 2. At the time of the electrophysiological study, most of the patients (58/63, 92%) were in AFL, including a patient coexisting with third‐degree atrioventricular block, and the remaining patients (5/63, 8%) were in sinus rhythm but the clinical AFL were induced successfully before mapping. Electrophysiological mapping and entrainment pacing confirmed that all the AFLs were CTI‐dependent with entire TVA involvement. Compared with the nonsurgical group, the RA volume measured by electroanatomical mapping was significantly larger (114.8±26.0 vs. 97.5 ± 19.1 mL, *p* = 0.004) and the mean AFL cycle length (CL) was much longer (253.3 ± 40.4 vs. 234.1 ± 24.2 ms, *p* = 0.03) in the postsurgical group.

**Table 2:** Electrophysiological and ECG characteristics in relation to AFL.

Parameters	Postsurgical group (*n* = 21)	Nonsurgical group (*n* = 42)	*p* Value
AFL cycle length, ms	253.3 ± 40.4	234.1 ± 24.2	0.03
RA volume, mL	114.8 ± 26.0	97.5 ± 19.1	0.004
Mechanism of reentry			
CCW direction (*n*, %)	18 (85.7%)	36 (85.7%)	1.0
CW direction (*n*, %)	3 (14.3%)	6 (14.3%)	1.0
ECG characteristics			
Typical ECG for CCW	10 (55.6%)	31 (86.1%)	0.01
Typical ECG for CW	1(33.3%)	4(66.7%)	0.34

**Figure 2:**
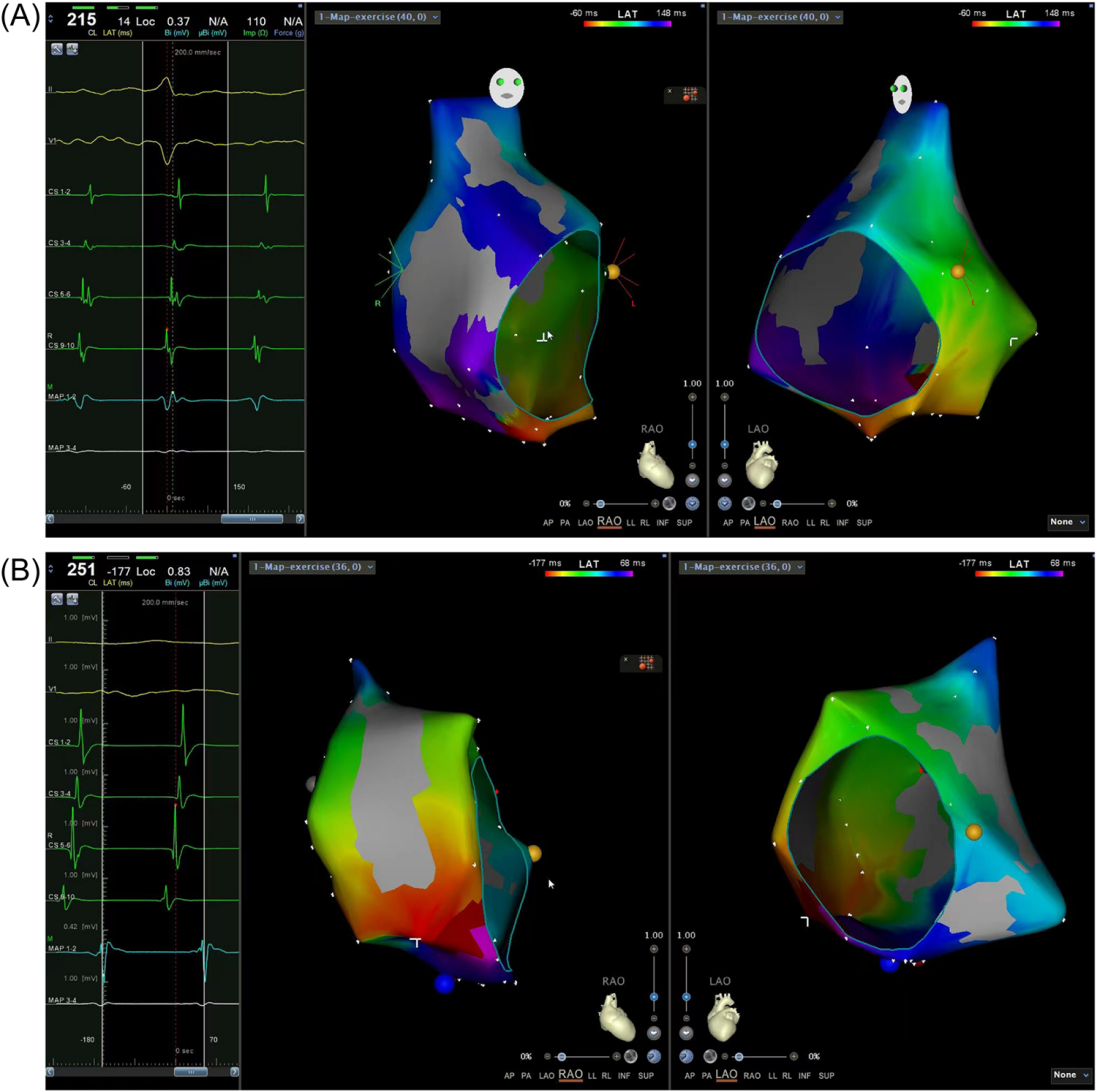
(A) illustrates an activation pattern as CCW‐AFL with a short cycle length of 220 ms in a nonsurgical patient, confirmed by electrophysiological mapping. (B) Shows an activation pattern as CW‐AFL with a much longer cycle length of 260 ms in a postsurgical patient. AFL, atrial flutter; CCW, counterclockwise; CW, clockwise.

In the postsurgical group, 85.7% (18/21) of the patients were diagnosed with CCW‐AFL and 14.3% (3/21) CW‐AFL confirmed by electrophysiological mapping. However, only 55.6% (10/18) of postsurgical patients with proven CCW‐AFL presented with an ECG morphology typical for this mechanism, showing the characteristic “sawtooth” pattern in the inferior leads without isoelectric lines and positive flutter wave in lead V_1_ (Figure 1A), while the remaining 8 patients demonstrated atypical ECG patterns, the orientation in the inferior leads differed considerably (Figure 1C). Of the three patients with CW‐AFL, one presented with a typical ECG morphology for the mechanism, positive in the inferior leads and negative in lead V_1_ (Figure 1B). The remaining 2 patients had atypical ECG patterns, with low and flat flutter waves and significant isoelectric lines measured between flutter waves in the inferior leads, negative in lead V1 (Figures 1D and 2B).

In the nonsurgical group, CCW‐AFL was revealed in 85.7% (36/42) of the patients and 14.3% (6/42) had CW‐AFL confirmed by electrophysiological mapping. Additionally, 86.1% (31/36) of the patients proven CCW‐AFL presented a typical ECG pattern for this mechanism, positive in the inferior leads and negative in lead V_1_ (Figures 1A and 2A), and the remaining 13.9% (5/36) patients had atypical ECG morphologies (Figure 1C). Of the 6 patients with CW‐AFL, four patients proven CW‐AFL presented with a typical ECG morphology, showing positive flutter waves in the inferior leads and negative flutter wave in lead V_1_ (Figure 1B) and the remaining two patients had atypical ECG patterns (Figure 1D).

### 3.3 Ablation procedure

**Figure 3:**
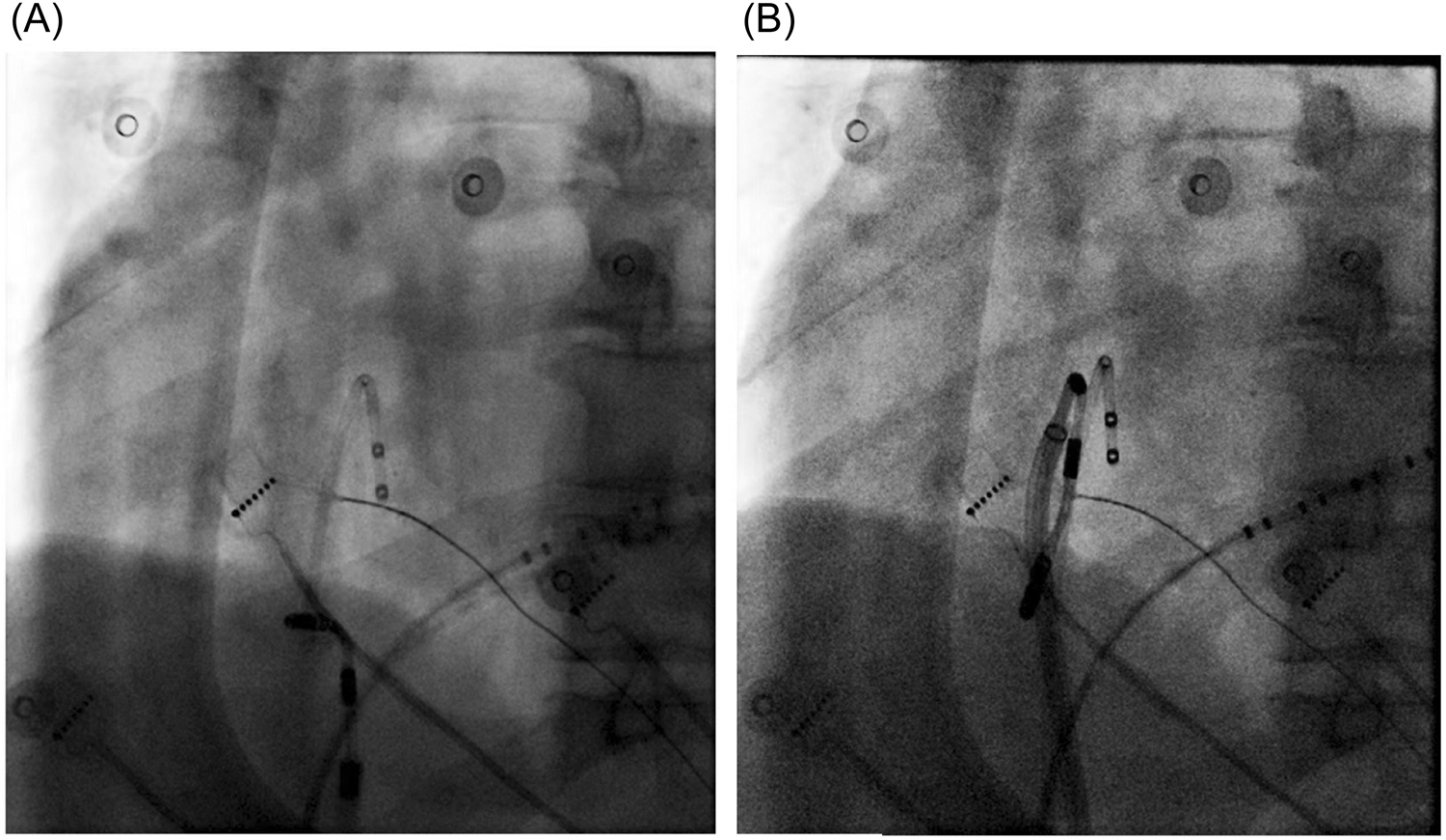
(A) Represents an example of CTI ablation, a linear lesion line drawn between the tricuspid annulus (TA) and the inferior vena cava (IVC) at six o'clock direction from left anterior oblique view and the ablation catheter was close to the ventricular side. (B) illustrates that a reverse “U‐curve” approach is applied to achieve more stable contact when ablating in proximity to the IVC ostium. CTI, cavo‐tricuspid isthmus.

The results of the ablation procedure are presented in Table 3. In the present study, all the AFL were successfully eliminated and bidirectional CTI block were achieved in both groups. We routinely employed a reverse “U‐curve” to facilitate RF delivery guided by RMN, especially when ablating in proximity to the IVC ostium (Figure 3). The patient coexisting with 3° atrioventricular block received a dual‐chamber pacemaker implantation after catheter ablation. No minor or major complications occurred during the procedures. Compared with the nonsurgical patients, the total procedure time (75.9 ± 21.3 vs. 61.6±26.6 minutes, *p* = 0.03), RF time (18.7 ± 7.2 vs. 13.6 ± 8.0 minutes, *p* = 0.04) and ablation time (53.0 ± 21.4 vs. 36.7 ± 25.6 minutes, *p* = 0.02) were much longer in the postsurgical patients. Additionally, the navigation index in the postsurgical patients was significantly smaller (0.35 ± 0.08 vs. 0.43 ± 0.13, *p* = 0.01), suggesting that it is more difficult to reach the target locations and maintain good catheter stability compared with the nonsurgical patients. However, there was no significant difference between the groups with regard to fluoroscopy time (2.1 ± 0.5 vs. 2.3 ± 1.3 minutes, *p* = 0.51).

**Table 3:** Procedural parameters.

Parameters	Postsurgical group	Nonsurgical group	*p* Value
Procedure time, mins	75.9 ± 21.3	61.6 ± 26.6	0.03
Clinical time, mins	66.3 ± 20.0	50.4 ± 25.8	0.02
Total X‐ray time, mins	2.1 ± 0.5	2.3 ± 1.3	0.51
RF applications, *n*	45.4 ± 15.3	30.9 ± 19.1	0.01
RF time, mins	18.7 ± 7.2	13.6 ± 8.0	0.04
Mapping time, mins	12.5 ± 4.2	11.7 ± 4.5	0.58
Ablation time, mins	53.0 ± 21.4	36.7 ± 25.6	0.02
Navigation index	0.35 ± 0.08	0.43 ± 0.13	0.01

Abbreviation: RF, indicates radiofrequency.

### 3.4 Follow‐up

After a mean follow‐up of 27 ± 14 months, no patients had recurrences of CTI‐dependent AFL in either group.

## 4 DISCUSSION

### 4.1 Main findings

The main findings are as follows: (1) Compared with the nonsurgical group, the mean AFL CL is significantly longer and the RA volume is larger in the postsurgical group; (2) The electrophysiological study reveals that the majority of AFL are CCW direction in both groups, however, ECG presentation may be atypical for the mechanism in the postsurgical AFL while most of the nonsurgical patients show a typical ECG pattern; (3) The acute and long‐term success rates of catheter ablation guided by RMN are high and comparable in both groups, but the procedure time, RF time and ablation time are much longer in the postsurgical group.

### 4.2 Difference in AFL cycle length

In the present study, we observed that nonsurgical AFL was more prevalent in men than in women, which is well in line with a previous study.^9^ Moreover, we have found that the postsurgical AFL CL is much longer than that of the nonsurgical AFL. AFL CL depends on conduction velocity and length of the reentry circuit. A previous study suggested that cardiac surgery was the strongest factor independently associated with longer AFL CL.^10^ The majority of these procedures involve RA cannulation or incisions resulting in RA scarring with a resultant slowing of atrial conduction. Further, larger RA volume were observed in the postsurgical patients, resulting in longer AFL circuits. Nevertheless, the shortest AFL CL (200 ms) observed was present in one patient after CABG. Therefore, short AFL CL does not preclude the possibility of CTI‐AFL even in case with prior cardiac surgery history.

### 4.3 Difference in ECG pattern presentation

The flutter wave morphologies on ECG have been well described in CTI‐AFL. Nevertheless, ECG findings might not be as helpful for identifying tachycardia mechanisms in patients with structurally abnormal hearts compared to those without structural heart disease.^11^ Typical ECG patterns of CCW AFL are well known as having a sawtooth appearance in the inferior leads and positive flutter waves in lead V_1_. Our analysis of the AFL ECG revealed that only 55.6% of patients with proven CCW AFL presented with a typical ECG morphology suggesting this mechanism. In contrast, 86.1% of nonsurgical patients demonstrate a typical ECG pattern reflecting the mechanism. Previous study has revealed that approximately 80% of patients without prior surgery presented with a typical ECG morphology for CCW AFL.^12^ The atypical flutter wave morphologies are largely determined by conduction in the RA and are also associated with interatrial connections and activation sequences of the LA.^12^ Hence, prior surgical atriotomies or atrial baffle procedures modify the RA conduction properties in the postsurgical patients, accounting for the atypical ECG morphologies. Moreover, long isoelectric lines between flutter waves in this study might reflect additional areas of slow conduction, rather than representing focal activity involving the RA.

### 4.4 Effectiveness of ablation therapy

CTI linear ablation is a standard practice for the treatment of CTI‐AFL, with high acute and long‐term success rates.^13^ The present study found that acute and long‐term successful outcome of CTI‐AFL ablation were high and comparable in both groups. Compared to previous reports^14^, ^15^ on RMN‐guided catheter ablation of CTI‐AFL in patients without cardiac surgery, the total procedure duration, ablation time and fluoroscopy time were much shorter, with higher acute and long‐term success rates in our study. There might be several explanations for the results. First, all the procedures were performed using the third‐generation RMN system (Niobe ES), whose software have been renewed simultaneously. Thus, the system can provide faster magnetic field direction changes, resulting in reducing procedure time during AF ablation. Second, the ablation catheter in previous studies was a 8 mm tip nonirrigated catheter, however, the catheter used in the present study was a 3.5 mm tip irrigated catheter, leading to higher RF efficacy as well as better acute and long‐term outcomes. Third, the conduction gaps during CTI ablation are mostly close to the IVC ostium. We routinely employ a reversed U curve guided by RMN to facilitate the RF delivery in proximity to IVC, which is helpful to achieve bidirectional CTI block.

In addition, the procedure and ablation times were much longer in the postsurgical patients compared with the nonsurgical patients. Despite sharing the same tachycardia circuit, several factors could account for the differences on procedure parameters. The ability to easily access the tachycardia circuit with the ablation catheter can be complicated by anatomical barriers caused by surgical repair, especially in the setting of tricuspid valve surgery,^16^ since the navigation index was much smaller in the postsurgical group. Moreover, the presence of fibrosis or markedly enlarged RA may have a negative impact on the transmurality of the ablation lesions.^17^

### 4.5 Empirical CTI ablation and prophylactic AF ablation

In our study, all the CTI‐AFLs were induced successfully during mapping. In routine practice, empirical CTI ablation was adopted if the tachycardia was judged as typical CTI‐AFL by ECG analysis before procedure. Sawhney et al.^18^ has shown that long‐term outcomes (over 7 years) after empiric and entrained CTI ablation for CTI‐AFL are comparable. Hence, an empiric CTI line should be considered in the case of noninducibility of CTI‐AFL.

Previous study has suggested that AF incidence is markedly elevated in AFL patients compared to the general population during long‐term follow‐up.^19^ However, we did not perform the prophylactic AF ablation in clinical practice. There are two patients developing AF and received AF ablation during follow‐up. Currently AHA/ACC Guidelines do not recommend the prophylactic approach, probably due to the high complexity and potential life‐threatening complications of this procedure from transseptal puncture required for AF ablation. Recent meta analysis has indicated the efficacy and safety of prophylactic pulmonary vein isolation (PVI) during CTI ablation in typical AFL patients without AF history, especially for elder patients.^20^ Mohanty et al.^21^ demonstrated that only patients ≥ 55 years having CTI + PVI showed significantly higher success compared to CTI only. Therefore, large randomized and prospective trials in the future are warranted to confirm the benefit of prophylactic AF ablation in typical AFL.

### 4.6 Limitation

Several limitations should be mentioned for the present study. First, this study is a retrospective study in a single center and has a relatively small size in the postsurgical group. Hence, extrapolating the present findings to the generalized population with previous cardiac surgery should be made with caution. The findings should be tested in prospective, large‐scale multicenter trials in the future. Second, only clinically symptomatic recurrences were recorded and monitoring to detect nonsymptomatic episodes was not conducted, so the real incidence of recurrences might have been underestimated.

## 5 CONCLUSION

Catheter ablation of CTI‐AFL guided by RMN with and without cardiac surgery are associated with high acute and long‐term success rates. Procedural and ablation times are much longer in the postsurgical patients than those of the nonsurgical patients. Moreover, ECG analysis reveals that ECG characteristics of the CTI‐AFL in the nonsurgical patients are mostly typical, while it may be misleading as they are more often atypical in the postsurgical patients. These findings might be in relation to the anatomical changes in the pathophysiological substrate of CTI‐AFL.

## CONFLICT OF INTEREST STATEMENT

The authors declare no conflict of interest.

## Data Availability

The data that support the findings of this study are available on request from the corresponding author. The data are not publicly available due to privacy or ethical restrictions.
